# The heavy burden and treatment challenges of fungal periprosthetic joint infection: a systematic review of 489 joints

**DOI:** 10.1186/s12891-024-07616-6

**Published:** 2024-08-16

**Authors:** Guangqian Shang, Siqi Zhao, Shuai Yang, Ji Li

**Affiliations:** 1 Department of Orthopedics, The Fourth Medical Center of Chinese People’s Liberation Army General Hospital, No.51 Fucheng Road, Haidian District, Beijing, China; 2 School of Medicine, Nankai University, No.94 Weijin Road, Nankai District, Tianjin, China; 3 Operation Room, The People’s Hospital of Linqing, No.317 Xinhua Road, Linqing District, Liaocheng, Shandong China; 4 Institute of Orthopedics, Third Hospital of Hebei Medical University, No.139 Ziqiang Road, Qiaoxi District, Shijiazhuang, Hebei China

**Keywords:** Fungal periprosthetic joint infection, Joint, Two-stage exchange, Treatment, Recurrence

## Abstract

**Background:**

Fungal periprosthetic joint infection (FPJI) is an infrequent but devastating complication that imposes a heavy burden on patients. At present, a consensus regarding the most optimal surgical option for patients with FPJI, the ideal duration of systemic antifungal treatment, and many other issues has not been reached.

**Methods:**

A comprehensive literature search was performed on the PubMed and Embase databases. The search criteria employed were as follows: (fungal OR candida OR mycotic) AND periprosthetic joint infection. Initially, the titles and abstracts were screened, and subsequently, studies deemed irrelevant or duplicative were eliminated. Following this, the complete texts of remaining articles were thoroughly examined. According to the inclusion and exclusion criteria, 489 joints in 24 articles were screened out. We further extracted the demographic characteristics (age, gender, body mass index, etc.), clinical presentation, fungal species, presence of bacterial coinfection, surgical methods, systemic and local antifungal therapy, and treatment outcomes. Subgroup data were analyzed according to fungal species and bacterial coinfection. Univariate logistic regression analysis was conducted to ascertain the risk factors associated with the infection recurrence.

**Results:**

A total of 506 fungi were identified within 489 joints. The most prevalent fungal species were Candida albicans (41.5%). Out of 247 joints (50.5%) presenting with concurrent fungal and bacterial infections. Among the initial surgical interventions, two-stage exchange was the most common (59.1%). The infection recurrence rates of DAIR, resection arthroplasty, two-stage, one-stage, and three-stage exchange were 81.4%, 53.1%, 47.7%, 35.0%, and 30%, respectively. The mean duration of systemic antifungal therapy was 12.8 weeks. The most common drugs used both in intravenous (55.9%) and oral therapy (84.0%) were fluconazole. The proportion of patients who used antifungal drugs after replantation (two-stage and three-stage) was 87.6%. 33.2% of cement spacer or fixed cement contained antifungal drugs, of which amphotericin B was the main choice (82.7%). FPJI caused by candida albicans (OR = 1.717, *p* = 0.041) and DAIR (OR = 8.433, *p* = 0.003) were risk factors for infection recurrence.

**Conclusions:**

Two-stage exchange remains the most commonly used surgical approach. The reliability of one- and three-exchange needs further evaluation due to the small sample size. Antifungal-loaded cement spacers, and direct intra-articular injections of antimycotics after reimplatation should be strongly considered. Medication is not standardized but rather individualized according to microbiology and the status of patients.

## Introduction

Periprosthetic joint infection (PJI), a devastating complication after total joint arthroplasty (TJA) with an incidence of 1%-4% worldwide, imposed serious physiological and psychological burdens on patients [1–3]. Fungal infections are infrequent, representing a mere 1% of all PJI occurrences [4]. Nevertheless, with the growing number of patients undergoing TJA in the next decade, according to the United States’ forecast, it is expected that the probability of encountering PJI will escalate concurrently [5]. As a result, the incidence of fungal PJI (FPJI) will also increase accordingly.

When compared to bacterial infections, acute FPJI is uncommon, and its clinical manifestation seems to be comparatively milder [6]. Preoperative systemic inflammatory markers, including C-reactive protein (CRP) and erythrocyte sedimentation rate (ESR), and synovial fluid cell count analyses from joint aspirations cannot differentiate bacterial from fungal PJI [7]. So early and accurate detection of fungal PJI is needed, mitigating the detrimental effects of delaying treatment. Because of the prolonged culture periods and the requirement for special culture mediums, it is difficult to isolate fungi in culture, and sometimes false negative results appear, which leads to the failure of revision surgery. Metagenomic next-generation sequencing (mNGS) may be able to resolve this problem and improve diagnostic efficiency [8].

Several potential risk factors linked to the onset of FPJI have been identified, including immunosuppression, inappropriate antibiotic usage, diabetes mellitus, multiple revision surgeries and so on [9–11]. The rate of treatment failure for FPJI is more than twofold greater than for bacterial PJI [9]. The close association between this high failure rate and the distinctive biological behavior of fungi is evident, such as a high degree of adaptability to dynamic environments, effective adhesion to human hosts, and the ability to form a drug-resistant biofilm layer [12]. Moreover, at present, there are no standardized or international protocols to guideline the treatment of FPJI, especially the choice of surgical approach, which is also one of reasons for the poor prognosis.

This systematic review was performed to 1) evaluate demographic characteristics, fungal species, treatment, and prognosis of patients with FPJI; 2) analyze differences between groups, including infection caused by Candida albicans (CA) or non-CA, and presence or absence bacterial coinfection; 3) explore potential risk factors for infection recurrence. It aimed to provide specific evidence-based guidance to clinicians, which had more important significance for inexperienced grass-roots hospitals.

## Material & methods

### Article selection

The systematic review was conducted according to the Preferred Reporting Items of Systematic Reviews guidelines [13]. A comprehensive literature search was performed on March 15, 2024, utilizing the PubMed and Embase databases. The search criteria employed were as follows: (fungal OR candida OR mycotic) AND periprosthetic joint infection. A combined total of 1,297 articles were retrieved from the PubMed database, while the Embase database yielded 280 articles. To maintain precision, the process of literature screening was carried out by two autonomous evaluators. Initially, the titles and abstracts were screened, and subsequently, studies deemed irrelevant or duplicative were eliminated. Following this, the complete texts of remaining articles were thoroughly examined, and the assessment of their eligibility was carried out in accordance with the predetermined inclusion and exclusion criteria. In addition, the references to the chosen articles were reviewed to identify any pertinent sources that might have been omitted during the retrieval procedure.

The inclusion criteria that were taken into account are as follows: (1) be published in English and in peer-reviewed journals; (2) be diagnosed as FPJI meeting the major or minor criteria of Musculoskeletal Infection Society (MSIS) [14]; (3) provide accurate and comprehensive data, encompassing demographic characteristics, treatment methodologies and other pertinent information; (4) not less than 6 months of follow-up period. Additionally, expert opinions, book chapters, case reports, literature reviews, meta-analyses, letters to the editor, cadaver or in vitro investigations, and animal model studies were excluded.

### Quality assessment

As the included studies in this review were observational studies, we evaluated their quality employing the “Strengthening the Reporting of Observational Studies in Epidemiology” statement described by Summers et al. [15, 16]. Items included setting, participants, variables, data sources, statistical methods, participants, descriptive data, outcome data, main results, and limitations. Each item was scored as poorly described (0 points), partly described (1 points), and well described (2 points). Articles with a total score of > 15 points were deemed eligible for inclusion.

### Data extraction

Demographic attributes from the selected studies included age, gender, body mass index (BMI), affected joint, American Society of Anaesthesiologists (ASA) score, Charlson Comorbidity Index (CCI), and preoperative affected joint scores. Focused on infection for this systematic review, we extracted the time elapsed between initial surgery and symptom onset, hematological index, clinical presentation, fungal pathogens, presence of bacterial coinfection, antifungal regimen (both pharmacological and surgical management), follow-up period and treatment outcomes. Surgical methods included debridement, antibiotics, and retention of the implant (DAIR), resection arthroplasty, arthrodesis, amputation, one-stage, two-stage, and three-stage exchange. Furthermore, when it comes to the cement spacer, we conducted an evaluation to determine whether antifungal-agent-loaded cement was used, or antifungal agents were incorporated into delivery pellets such as calcium sulfate. The main outcome assessed in this study was the rate of infection recurrence following each surgical treatment. Another concern was the successful treatment rate at the last follow up. According to the Delphi consensus criteria [17], it was defined the following: (1) all manifestations and indications of infection disappeared, including clinical, radiological, and laboratory signs, such as a healed wound without fistula, drainage or pain; (2) no further procedures, e.g., the use of suppressive antibiotics, or surgical interventions; (3) no occurrence of PJI-related mortality; (4) with a functioning prosthesis in situ.

### Statistical analyses

Due to the heterogeneity of the articles, a meta-analysis could not be conducted. All statistical analyses were performed using SPSS (version 24.0; IBM Corp., Armonk, NY, USA). According to different groups of fungal species and bacterial coinfections, demographic, clinical, and treatment data were analyzed using descriptive statistics. Continuous variables were expressed as means and standard deviations (SDs). Mann–Whitney test was used to compare continuous variables between the two groups. Categorical variables were presented as frequencies and constituent ratios and were compared using chi-squared test. Due to the limitation of data extracted from the articles, only univariate logistic regression analysis was conducted to ascertain the risk factors associated with the infection recurrence. *P* < 0.05 indicated statistical significance.

## Results

### Study population

**Fig. 1:**
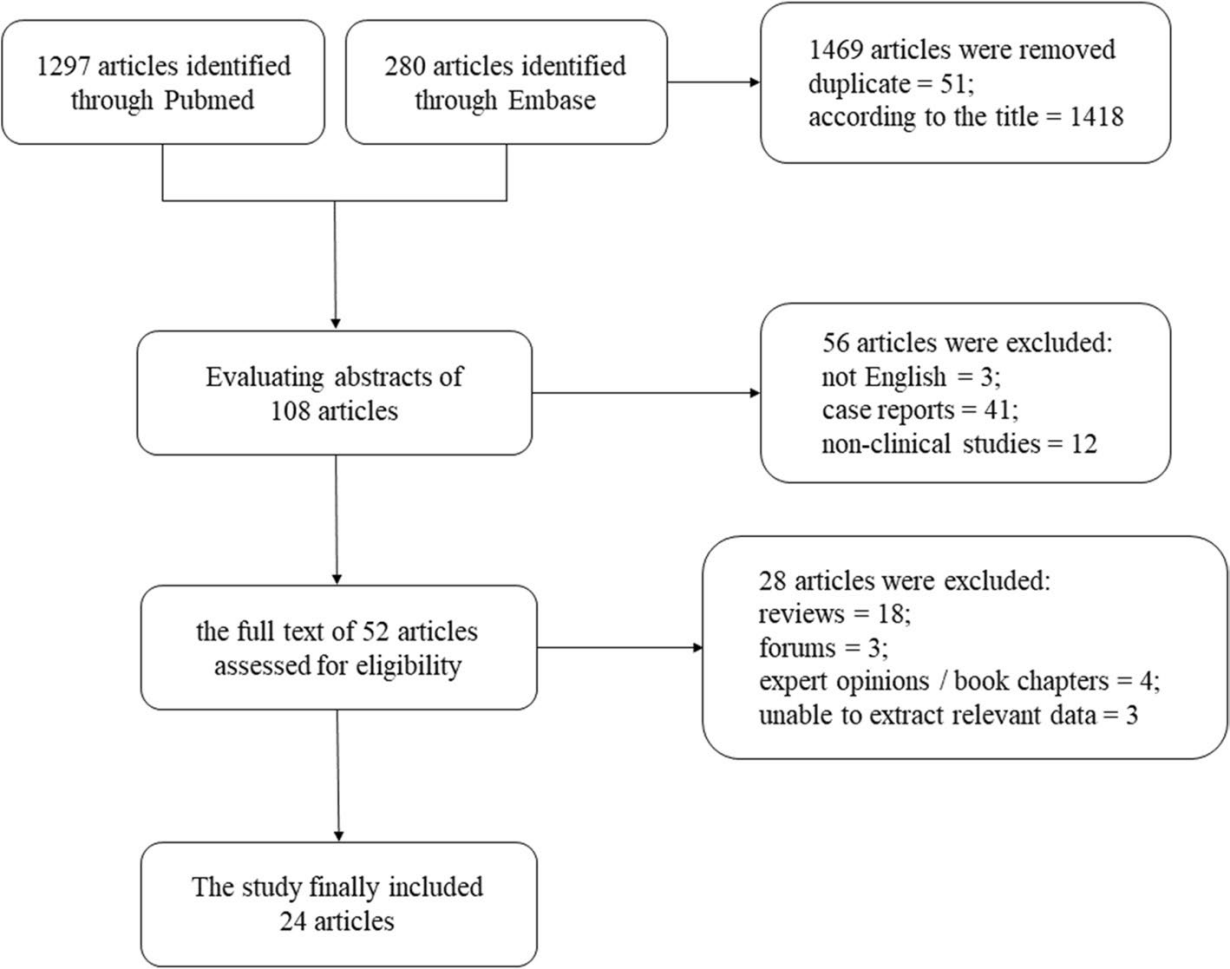
PRISMA (Preferred Reported Item for Systematic review and Meta-Analysis) flow diagram of the articles extracted from databases

According to the inclusion and exclusion criteria, this comprehensive review yielded a total of 24 clinical studies pertaining to FPJI, reported between 1989 and 2023 (Fig. 1). According to the results of quality assessment, these articles met the criteria. The retrieved data encompassed 484 patients, predominantly sourced from cohort studies featuring larger sample sizes (16 articles), with a limited number originating from small case series (8 articles). And a total of 489 joints were involved, comprising 302 knees and 187 hips. Five patients were diagnosed with bilateral FPJI. The mean age and BMI observed in the collective studies were 67.97 years and 28.09 kg/m^2^, respectively, with a total of 198 males and 286 females. Ten articles, encompassing 150 patients, did not provide information regarding BMI. Additional demographic and clinical information could be found in the Table 1.

**Table 1:** Demographic characteristics, treatment, and prognosis of patients diagnosed as fungal prosthetic joint infection in literature

Study (year)	Number of patients (n)	Gender (n)	Joint (n)	Mean age (years)	Mean BMI (kg/m^2^)	Mean time from TJA to FPJI (m)	Mean FU (m)	Mean CRP* (mg/L)	Mean ESR* (mm/h)	Fungal strain (n)	Mixed infection with bacteria (n)	Surgical treatment (n)	Treatment success at the last FU (n, rate)
Darouiche (1989) [18]	4	3 M 1F	1 K 3H	63.5	NR	4.8	12.6	NR	NR	4CA	0	1 DAIR 3 RA	0, 0%
Azzam (2009) [19]	31	14 M 17F	17 K 14H	64	29.1	25	45	17.5	54	23CA 8NCA 3NCF	5	7 DAIR 24 two-stage	13, 41.9%
Dutronc (2010) [20]	7	3 M 4F	4 K 3H	72	NR	NR	30	98.1	NR	4CA 3NCA	0	1 DAIR 1 arthrodesis 1 RA 1 no surgery 3 two-stage	2, 28.6%
Anagnostakos (2012) [21]	7	5 M 2F	3 K 4H	68	NR	NR	28	NR	NR	3CA 4NCA 1NCF	0	7 two-stage	5, 71.4%
Hwang (2012) [22]	28	1 M 27F	30 K	69	NR	19.6	51.6	43	39	7CA 17NCA 6NCF	6	4 DAIR 26 two-stage	29, 96.7%
Kuiper (2013) [23]	8	2 M 6F	8H	72.8	NR	13.1	30.4	47	72.2	7CA 2NCA	8	1 RA 3 DAIR 4 two-stage	2, 25%
Ueng (2013) [11]	16	12 M 4F	9 K 7H	62	27	21	41	NR	NR	9CA 7NCA	8	16 two-stage	8, 50%
Klatte (2014) [24]	10	6 M 4F	4 K 6H	68	NR	NR	84	22	NR	5CA 5NCA	1	10 one-stage	10,100%
Wang (2015) [25]	5	2 M 3F	5 K	67	NR	7.4	41.6	22.8	40.2	5NCA	0	5 two-stage	5, 100%
Geng (2016) [26]	8	3 M 5F	4 K 4H	60	22.9	21.6	52.8	46.8	68	3CA 3NCA 2NCF	5	1 RA 7 two-stage	7, 87.5%
Ji (2017) [27]	11	4 M 7F	7H 4 K	60.6	23.6	NR	60	NR	NR	6CA 5NCA	3	11 one-stage	11, 100%
Brown (2018) [28]	31	14 M 17F	18 K 13H	68.6	30.3	NR	48.8	53	NR	15CA 10NCA 6NCF	6	1 one-stage 4 DAIR 5 RA 5 no surgery 16 two-stage	13, 76.5% (Only 17 were evaluable)
Gao (2018) [29]	17	7 M 10F	13 K 5H	61.1	25.1	NR	65.1	44	57.5	4CA 11NCA 4NCF	9	5 DAIR 13 two-stage	16, 88.9%
Kuo (2018) [9]	29	14 M 15F	15 K 14H	70.7	34.2	14.6	100.8	6.6	59.2	7CA 11NCA 9NCF 2NR	15	7 DAIR 19 two-stage 3 one-stage	14, 48.3%
Kim (2018) [30]	9	1 M 8F	9 K	76	26.7	20	66	22.5	56	9NCA	2	9 two-stage	9, 100%
Theil (2019) [4]	26	NR	8 K 18H	72.5	29.5	NR	33	NR	NR	19CA 8NCA	13	2 one-stage 24 two-stage	12, 46.2%
Sidhu (2019) [31]	22	13 M 9F	14 K 8H	64.5	30.9	36.4	49.2	62.1	47.6	10CA 9NCA 4NCF	22	7 DAIR 15 two-stage	9, 40.9%
Saconi (2020) [32]	11	5 M 6F	5 K 6H	65.1	NR	43.4	41.7	312	34.8	6CA 6NCA 1NCF	6	1 DAIR 1 two-stage 3 one-stage 4RA	4, 44.4% (Only 9 were evaluable)
Baecker (2021) [33]	18	8 M 10F	7 K 11H	72.8	27.9	NR	35	NR	NR	7CA 10NCA 1NCF	7	18 three-stage	13, 72.2%
Grzelecki (2022) [34]	8	3 M 5F	6 K 2H	72.5	30.7	58.4	17.6	24.9	50.8	3CA 5NCA	1	1 one-stage 7 two-stage	4, 50%
Karczewski (2022) [35]	29	13 M 16F	15 K 14H	71	NR	NR	33	51.7	NR	17CA 13NCA 1NCF	22	1 hemipelvectomy 2 DAIR 2 one-stage 4 RA 8 two-stage 12 three-stage	18, 62.1%
Herndon (2023) [36]	41	23 M 18F	22 K 19H	64	NR	NR	12	NR	NR	20CA 21NCA 3NCF	37	1 one-stage 2 arthrodesis 2 amputation 2 ID 4DAIR 4 two-stage 26 RA	NR
McCulloch (2023) [37]	67	32 M 35F	45 K 24H	68	30.8	NR	34	NR	NR	28CA 29NCA 4NCF 8NR	61	5 one-stage 16 DAIR 48 two-stage	29, 42.0%
Yang (2023) [38]	41	10 M 31F	41 K	77.6	24.5	NR	NR	7.9	60.5	2CA 39NCA	13	10 DAIR 31 two-stage	26, 63.4%

*FPJI* Fungal prosthetic joint infection, *BMI* body mass index, *TJA* total joint arthroplasty, *FU* follow up, *CRP* C-reactive protein, *ESR* erythrocyte sedimentation rate, *NR* not reported, *CA* candida albicans, *NCA* non-candida albicans, *NCF* non-candida fungi, *DAIR* debridement, antibiotics, and implant retention, *RA* resection arthroplasty, *ID* irrigation and debridement alone

^*^ At the time of diagnosis

#### Fungal species

**Fig. 2:**
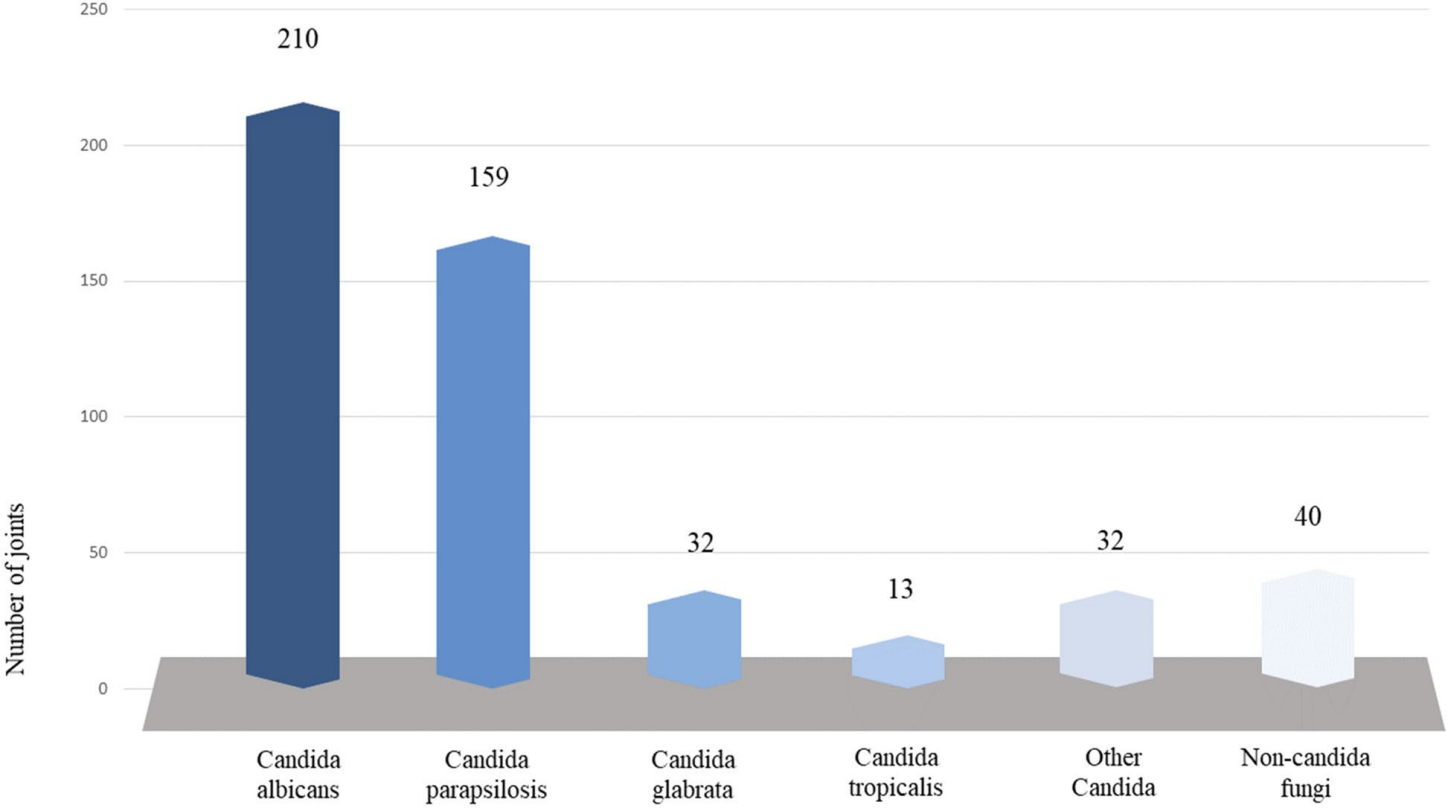
The fungal species of Fungal prosthetic joint infection

A total of 506 fungi were identified within 489 joints. Of the isolated fungal pathogens, the three most prevalent species were CA, Candida parapsilosis and Candida glabrata, with reported occurrences of 210 (42.9%), 159 (32.5%) and 32 (6.5%) joints, respectively (Fig. 2). Other Candida species (9.4%) that were less frequently observed included Candida tropicalis (14), Candida pelliculosa (6), Candida dubliniensis (5), Candida famata (5), Candida guilliermondii (4), Candida lusitaniae (3), Candida krusei (2), Candida freyschussii (2), Candida rugosa, Candida orthopsilosis, Candida lipolytica, Candida pseudotropicalis, and Candida utilis, each present in one joint. Forty joints (8.2%) were identified non-candida fungi infection: Aspergillus (19), Rhodotorula minuta (4), Pichia anomala (4), Alternaria (3), Penicillium (2), Trichosporon asahii (1), Scedosporium (1), Pithomyces (1), Aureobasidium (1), Acremonium strictum (1), Blastoschizomyces capitatus (1), Verticillium (1), and Phialemonium curvatum (1). Specific pathogenic organism was not reported in 20 joints. There were 16 patients (16 joints) with FPJI caused by a least two fungi: CA and Candida parapsilosis (5), CA and Candida glabrata (3), CA and Aspergillus (2), Candida parapsilosis and Candida tropicalis (1), Candida glabrata and Candida krusei (1), CA, Candida orthopsilosis and Trichosporon asahii (1), and unknown specific pathogens (3).

#### Bacterial coinfection

**Fig. 3:**
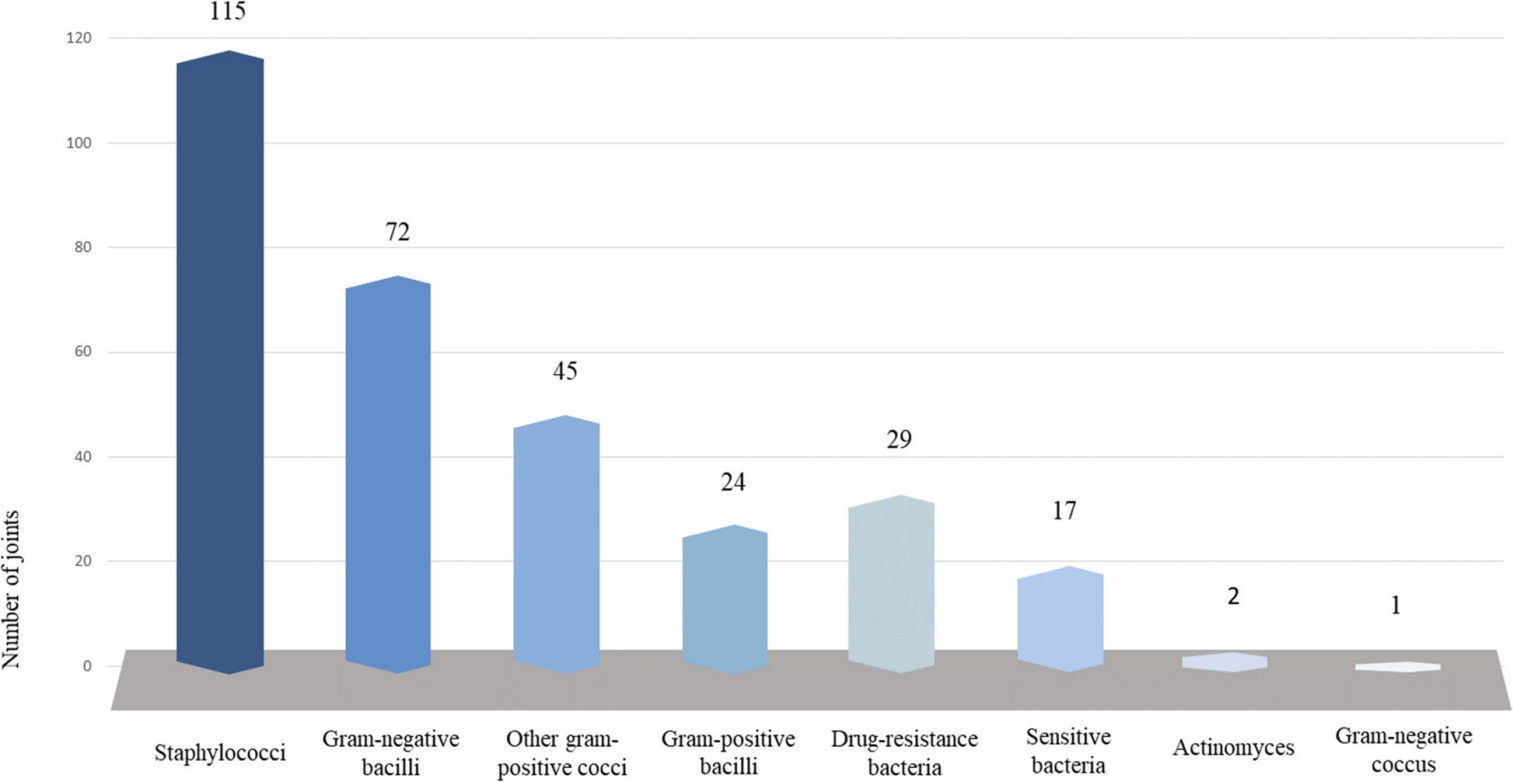
The bacterial species of prosthetic joint infection caused by bacterial and fungal co-infection

Out of 247 joints (50.5%) presenting with concurrent fungal and bacterial infections, 303 bacterial organisms were detected (Fig. 3). Staphylococcus (115) and Gram-negative Bacillus (72) were the predominant causative agents of bacterial infections. Among the former, coagulase-negative staphylococcus (43), Staphylococcus aureus (35) and Staphylococcus epidermidis (24) were the most common, whereas Escherichia coli (13), Pseudomonas aeruginosa (13) and Klebsiella pneumoniae (11) were the prevailing species within the latter. In addition, non-Staphylococcus gram positive coccus, gram-positive bacilli, drug-resistance bacteria, and sensitive bacteria were reported in 45, 22, 29 and 17 joints, respectively.

#### Surgical intervention

**Fig. 4:**
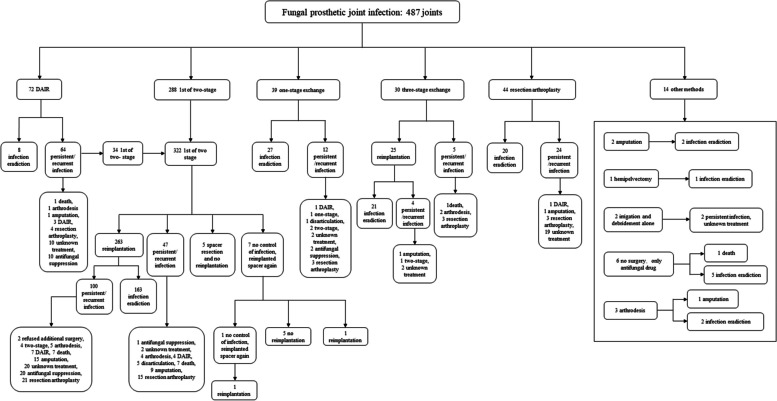
The flow chart outlines the surgical treatment of fungal prosthetic joint infection in 487 joints

The Fig. 4 outlined the surgical treatment of FPJIs in 487 joints (two joints were lost to follow-up). Among the initial surgical interventions, two-stage exchange was the most common surgical intervention at 59.1% (288/487), followed by DAIR at 14.8% (72/487), resection arthroplasty at 9.0% (44/487), one-stage exchange at 8.0% (39/487), and three-stage exchange at 6.2% (30/487). The whole treatment process for these patients was further comprehensively analyzed, revealing infection recurrence rates of 81.4% (70/86) for DAIR, 53.1% (26/69) for resection arthroplasty, 47.7% (155/325) for two-stage exchange, 35.0% (14/40) for one-stage exchange, and 30.0% (9/30) for three-stage exchange. At the last follow up, treatment outcomes for certain or all joints across the three studies could not be extracted (57 joints in total), thereby yielding a final treatment success rate of 60.2% (260/432) for FPJI.

#### Systemic and local antifungal therapy

Following the surgical intervention, patients proceeded to undergo a course of systemic antifungal therapy. Not all studies have reported the specific time and/or route of administration. The mean duration of systemic antifungal therapy was 12.8 weeks (range 1–104) among 276 patients. In intravenous therapy, fluconazole was the most widely used, accounting for 55.9% (100/179), followed by caspofungin (23.5%, 42/179) and amphotericin B (14.5%, 26/179). And fluconazole continued to be the preferred option for oral therapy (84.0%, 126/150). Of the 262 patients whose route of administration was not reported, three frequently employed antifungal agents were fluconazole (69.1%, 181/262), amphotericin B (19.5%, 51/262), and caspofungin (5.7%, 15/262). Among the patients who underwent either two-stage or three-stage procedures, 185 patients documented whether they used antifungal drugs after replantation, and the proportion of drug use was as high as 87.6% (162/185). Caspofungin was administered intravenously for a duration of 4 weeks in two patients, while Voriconazole was administered intravenously for a duration of 2 weeks in one patient. Fluconazole was administered to the remaining 159 patients through oral, intravenous, or combined routes, with an average period of 9.9 (range 1–24) weeks.

We collected data from 226 patients who implanted bone cement spacers, out of which 75 patients (33.2%) used spacers containing antifungal agents. The predominant selection was amphotericin B, which was used by 62 patients (average 0.40 g per 40 g of bone cement), voriconazole by 11 patients (average 0.75 g per 40 g of bone cement) and fluconazole by 2 patients (not report).

#### Subgroup data analysis

Due to nearly 50% prevalence of CA-induced infection among patients with FPJI, we have categorized them into two subgroups, namely group A (CA infections) and group B (non-CA infections), which aims to investigate potential disparities between the two groups (Table 2). No statistical differences were observed in demographic and preoperative clinical characteristics, except for affected joints and CRP levels (*P* = 0.000, *P* = 0.002, respectively). Concurrent non-CA fungi and bacterial infections were significantly less frequent and demonstrated statistical significance (*p* = 0.030).A notable disparity was observed in the use of antifungal in spacer or cement (*p* = 0.011). It was noteworthy that group A exhibited a higher percentage of recurrence (*p* = 0.041).

**Table 2:** Subgroup data analysis according to fungal species and bacterial coinfection

	Fungal species			Bacterial coinfection		
	Group A (*n* = 117)	Group B (*n* = 146)	*P* value	Group C (*n* = 160)	Group D (*n* = 83)	*P* value
Age (years)	67.35 ± 13.51	68.55 ± 10.44	0.792	67.59 ± 10.67	68.76 ± 13.69	0.144
Gender			0.932			0.213
Male	35 (38.89%)	45 (39.47%)		46 (35.94%)	34 (44.74%)	
Female	55 (61.11%)	69 (60.53%)		82 (64.06%)	42 (55.26%)	
BMI (kg/m2)	23.77 ± 4.35	26.92 ± 4.93	0.057	25.86 ± 4.30	25.48 ± 5.89	0.567
Affected joint			**0.000**			**0.000**
Knee	50 (42.74%)	109 (74.66%)		111	38	
Hip	67 (57.26%)	37 (25.34%)		(69.38%)	(45.78%)	
				49 (30.62%)	45 (54.22%)	
Time from TJA to FPJI (months)	28.18 ± 45.26	18.98 ± 19.76	0.653	22.80 ± 35.03	23.36 ± 30.92	0.840
Prior surgeries on the affected joint	2.98 ± 1.97	2.79 ± 2.41	0.193	3.11 ± 2.56	2.54 ± 1.77	0.337
Preoperative joint score	48.00 ± 6.75	47.78 ± 9.65	0.947	48.67 ± 10.46	47.00 ± 7.32	0.799
CRP (mg/L)	45.35 ± 35.70	27.68 ± 39.66	**0.002**	33.08 ± 40.97	34.15 ± 35.98	0.422
ESR (mm/h)	58.88 ± 41.41	44.08 ± 26.62	0.243	39.65 ± 27.08	64.00 ± 34.85	**0.001**
ASA	2.14 ± 1.07	2.70 ± 0.76	0.158	2.54 ± 0.72	2.67 ± 1.37	0.575
CCI	4.08 ± 3.20	4.75 ± 3.57	0.833	4.00 ± 3.45	5.63 ± 3.07	0.182
Sinus tract			0.838			0.112
Presence	16	21		21 (30.00%)	16 (45.71%)	
Absence	28	40		49 (70.00%)	19 (54.29%)	
Fungal infection caused by CA						**0.019**
Yes				104 (65.00%)	41 (49.39%)	
No				56 (35%)	42 (50.61%)	
Concurrent fungal and bacterial infections			**0.030**			
Yes	41 (42.27%)	42 (28.77%)				
No	56 (57.73%)	104 (71.23%)				
Multi-fungal infections			0.088			0.561
Yes	7 (5.98%)	2 (1.37%)		4 (2.50%)	4 (4.82%)	
No	110 (94.02%)	144 (98.63%)		156 (97.50%)	79 (95.18%)	
Type of surgery			0.056			0.376
DAIR	11 (9.40%)	5 (3.43%)		10 (6.25%)	5 (6.02%)	
Resection arthroplasty	11 (9.40%)	7 (4.79%)		10 (6.25%)	8 (9.64%)	
One-stage	13 (11.11%)	16 (10.96%)		22 (13.75%)	7 (8.43%)	
Two-stage	63 (53.85%)	98 (67.12%)		99 (61.88%)	47 (56.63%)	
Three-stage	13 (11.11%)	18 (12.33%)		16 (10.00%)	15 (18.07%)	
Other	6 (5.13%)	2 (1.37%)		3 (1.87%)	1 (1.20%)	
Duration of prosthesis-free interval (weeks)	13.16 ± 22.63	14.47 ± 15.05	0.265	14.92 ± 19.02	12.62 ± 15.25	**0.025**
The length of systematic antifungal (weeks)	13.07 ± 11.90	18.55 ± 19.40	0.370	16.49 ± 16.42	15.90 ± 17.92	0.544
Use of antifungal after reimplantation			0.101			0.292
Yes	27 (77.14%)	72 (88.89%)		59 (81.94%)	40 (90.91%)	
No	8 (22.86%)	9 (11.11%)		13 (18.06%)	4 (9.09%)	
Use of antifungal in spacer or cement			**0.011**			0.766
Yes	10 (20.41%)	37 (42.05%)		32 (75.76%)	15 (32.61%)	
No	39 (79.59%)	51 (57.95%)		59 (24.24%)	31 (67.39%)	
Recurrence			**0.041**			0.485
Yes	46 (39.32%)	40 (27.40%)		47 (29.38%)	28 (33.73%)	
No	71 (60.68%)	106 (72.60)		113 (70.62%)	55 (66.27%)	
Follow-up (months)	34.69 ± 27.23	38.81 ± 25.86	0.245	38.02 ± 24.97	44.10 ± 29.08	0.317

Values are presented as the mean ± standard deviation or n (%)

Statistically significant values are identified in boldface

*BMI* body mass index, *TJA* total joint arthroplasty, *FPJI* fungal prosthetic joint infection, *CRP* C-reactive protein, *ESR* erythrocyte sedimentation rate, *ASA* American Society of Anaesthesiologists score, *CCI* Charlson Comorbidity Index (CCI), *CA* candida albicans, *DAIR* debridement, antibiotics, and implant retention

*Group A* infection caused by CA, *group B* infection caused by non-CA fungi, *group C* Absence bacterial coinfection, *group D* Presence bacterial coinfection

Likewise, since fungal and bacterial co-infections accounted for more than half of patients diagnosed with FPJI, they were divided into group C (only fungi infection) and group D (bacterial coinfection) to evaluate the impact of bacteria on the perioperative and postoperative progression. As shown in Table 2, significant differences were observed in affected joints, ESR levels, infection caused by CA, and the duration of prosthesis-free interval between groups C and D (*P* = 0.000, *P* = 0.001, *p* = 0.019, *p* = 0.025, respectively).

### Risk factors for recurrence

 In logistics regression analysis, it was observed that FPJI caused by CA significantly increased the risk of infection recurrence (OR = 1.717, *p* = 0.041) (Table 3). When comparing the hip joint to the knee joint, the latter was identified as a protective factor against infection recurrence (OR = 0.424, *p* = 0.001). Similarly, the use of antifungal agents after reimplantation and their application in spacer or cement were also determined to be protective factors (OR = 0.170, *p* = 0.002 and OR = 0.293, *p* = 0.020, respectively). In type of surgery, DAIR was significantly associated with an increased risk of infection recurrence (OR = 8.433, *p* = 0.003).

**Table 3:** Logistics regression analysis to identify risk factors for infection recurrence

	OR (95% CI)	*P* value
Age	0.999 (0.975–1.023)	0.921
Gender (female)	0.604 (0.331–1.104)	0.102
BMI	1.158 (0.986–1.359)	0.074
Affected joint (knee)	0.424 (0.250–0.718)	**0.001**
Time from TJA to FPJI	0.999 (0.995–1.002)	0.478
Prior surgeries on the affected joint	1.079 (0.924–1.261)	0.337
Preoperative joint score	0.997 (0.896–1.109)	0.957
ASA	4.284 (0.796–23.063)	0.090
CCI	1.483 (0.890–2.471)	0.130
Presence of sinus tract	1.360 (0.519–3.566)	0.532
Fungal infection caused by CA	1.717 (1.021–2.886)	**0.041**
Concurrent fungal and bacterial infections	1.224 (0.693–2.160)	0.486
Multi-fungal infections	0.578 (0.118–2.844)	0.500
Type of surgery		
DAIR	8.433 (2.106–33.769)	**0.003**
Resection arthroplasty	1.917 (0.507–7.244)	0.338
Two-stage	1.989 (0.765–5.172)	0.158
Three-stage	0.737 (0.198–2.740)	0.649
Other	2.300 (0.424–12.465)	0.334
Duration of prosthesis-free interval	1.026 (0.999–1.054)	0.057
The length of systematic antifungal	0.999 (0.967–1.032)	0.952
Use of antifungal after reimplantation	0.170 (0.056–0.520)	**0.002**
Use of antifungal in spacer or cement	0.293 (0.104–0.823)	**0.020**

*OR* odds ratio, *CI* confidence interval, *BMI* body mass index, *TJA* total joint arthroplasty, *FPJI* fungal prosthetic joint infection, *CRP* C-reactive protein, *ESR* erythrocyte sedimentation rate, *ASA* American Society of Anaesthesiologists score, *CCI* Charlson Comorbidity Index (CCI), *CA* candida albicans, *DAIR* debridement, antibiotics, and implant retention

Statistically significant values are identified in boldface

## Discussion

In 1979, a patient diagnosed with FPJI was initially reported by MacGregor et al.[39]. After the prosthetic removal and fusion of the knee, combined with the administration of amphotericin B-5-fluorcytosine, the infection was ultimately eradicated. In the subsequent decades, a limited number of relevant clinical studies have been successively documented, but owing to the rarity of FPJI, mainly case reports and small cohort studies. The diagnosis and treatment of FPJI were discussed at the 2018 International Consensus Meeting on Orthopedic Infection; however, the evidence levels were considered low or moderate, and the recommendations provided were vague [10]. Baecker et al. [33] suggested that the treatment principles of bacterial PJI should not be equated with those of FPJI. At present, a consensus regarding the most optimal surgical option for patients with FPJI, the ideal duration of systemic anti-fungal treatment, and many other issues has not been reached. This review extracted the demographic characteristics, fungal species, treatment, and prognosis of 484 patients diagnosed with FPJI. The objective was to analyze differences between different subgroups, identify the risk factors for recurrence, and provide more clinical evidence and guidance for Joint Surgeons.

### Diagnosis

According to the MSIS criteria [14], the role of serum and synovial marker levels in differentiating fungal from bacterial infections obtained completely opposite results in different studies [7, 9, 40]. So the conclusion regarding the diagnostic value of these markers could not be reached, thus suggesting their utilization solely as a reference indicator. Because of more stringent culture conditions and longer cluture periods of fungi in comparsion to bacteria, FPJI has sometimes been misdiagnosed as aseptic loosening, particularly in the absence of acute infection symptoms, which could finally lead to severe consequences for patients. In order to enhance the diagnostic efficacy, it was necessary to prolong the duration of culture to four weeks and inoculate more than four specimens on three different culture media, including synovial and tissue cultures [8, 22, 41]. Besides, the detection rate of mNGS surpassed that of microbial culture to a significant extent, neither fungi nor bacteria [8, 42, 43]. A study conducted by Zhang et al. [8] showed that all 12 patients with fungal osteoarticular infection tested positive for mNGS, while 5 of them tested negative for culture. He et al. [44] reported that the detection rate of fungi using mGNS in patients with FPJI was not noly nearly twice that of traditional microbial cultures, but also the detection time was shortened by more than half. Thus, when conditions permitted, mNGS could be a valuable supplemental approach to reduce misdiagnosis, missed diagnosis, or delayed diagnosis in cases of FPJI.

### Risk factors leading to FPJI

Some risk factors associated with FPJI have been identified, which could be divided into intrinsic host factors and external factors. Intrinsic factors were mostly related to an impaired immune response, including immunosuppression (neutropenia, corticosteroids or other immunosuppressive drugs, history of organ transplantation, and acquired immunodeficiency syndrome), malignancy, the use of antineoplastic agents, diabetes mellitus, rheumatoid arthritis, tuberculosis, cirrhosis, renal insufficiency, dialysis, candida colonization, while external factors included chronic or prolonged use of antibiotics, presence of indwelling intravenous or urinary catheters, parenteral hyperalimentation, malnutrition, severe burns, injection drug use, presence of wound drainage for more than five days, prior bacterial PJI, and multiple revision surgeries or abdominal surgeries [9, 19, 23, 26, 36, 40, 45]. The discovery of more potential risk factors to reduce the occurrence of FPJI at its root seemed more meaningful than the exploration of FPJI treatment. Identifying of patients with a higher risk profile enabled surgeons to perform more presice preoperative risk evaluations and targeted interventions to prevent FPJI.

### Surgical intervention

In recent decades, more and more medical institutions have begun to utilize one-stage exchange in the management of bacterial PJI, but few in FPJI remain [46]. A study conducted by George et al. reported that no superiority was demonstrated between a one- and two-stage exchange in hip PJI [39]. Klatte et al. [24] and Ji et al. [27] reported that the infection recurrence rates of one-stage exchange used in patients with FPJI were as low as 10% and 27.3%, respectively. The key factor in the eradiction of infection was postoperative direct intra-articular injections of fungus-sensitive IV antibiotics (an average of 18 days) to maintain high local antibiotic concentrations, although no antifungal drugs were incorporated into the fixed bone cement or joint cavity during the surgical procedure [27]. The fugal minimum inhibitory concentrations was 100–10,009, because of the more intricate structure of its bioflim [47]. This attractive strategy was first implemented in FPJI and had the potential to be applied to other surgical modalities to improve fungal clearance. Before then, a similar method were mainly used for bacterial PJI with great success in two-stage exchange, for which the surgical failure rate was only 4.7% [48]. What calls for special attention was that prior to proceeding with a one-stage exchange, it was crucial to possess a thorough comprehension of the pathogen's characteristics and its antibiotic sensitivity profile. Therefore, patients needed joint aspiration preoperatively, while also ensuring a minimum antibiotic-free period of two weeks to avoid biases in the culture outcome. The primary constraint of this surgical technique pertained to the limited sample size of patients involved, necessitating further clinical studies to confirm aforementioned results.

Hennessy et al. reported the first patient who eradicated FPJI by two-stage exchange.[49]. As the gold standard protocol for the treatment of bacterial PJI, two-staged exchange was also the most commonly used surgical method of fungal PJI, accounting for 59.1% in our study. While treatment outcomes were different, the majority of medical institutions preferred two-stage exchange to address FPJ, with the infection recurrence rates ranging from 0% to 52.6% [4, 9, 11, 19, 21–23, 25, 26, 29–31, 34, 37, 38]. In addition the difference in baseline characteristics of patients and sample size, the high variability in treatment results also was attributed to the discrepant doseage and duration of local and systemic antifungal antimycotics. In the study by Azzam et al. [19], 29 patients underwent two-stage exchange, of which 10 patients experienced infection recurrence after reimplantation, and 10 patients did not reimplant because of persistent infection. One of the significant contributing factors to the unfavorable outcome was that only five spacers contained antifungals. The restricted blood circulation to the cortical bone, coupled with the potential formation of a hematoma surrounding the prosthesis that hindered the blood supply to adjacent tissues, all prevented delivery of systemic antibiotics [50]. So infection was difficult to clear with the lack of application of topical antifungals. Based on the accumulated experience of surgeons and acceptable treatment outcomes in previous cases, we still recommended two-stage exchange as the first choice of surgical method.

As an improvement of two-stage exchange, three-stage exchange required a additional scheduled surgery between prosthesis removal and reimplantation that included meticulous debridement and exchange of spacer, in order to minimize the local burden of fungi and maintain continuous delivery of high local antimycotics concentrations. Due to the advantage of a extra debridement and spacer exchange, its infection recurrence rate was theoretically much lower than that of one- and two-stage exchange, but in fact it was just the opposite, only 30% according our analysis [33, 35]. The only reasonable explanation was the insufficient sample size. Furthermore, whether prolonged treatment would increase perioperative and postoperative risks and complications, such as side effects of drug and mortality, was one of our concerns. Fortunately, in the studies reported by Baecker et al. [33] and Karczewski et al. [35], these worrisome results were not demonstrated. This surgical method was not recommended as it made the treatment more cumbersome and lacked discernible benefit on two-stage exchange.

It was unadvisable to consider DAIR as an effective method for eliminating fungal infections, given the infection recurrence rates ranging from 50 to 100% (most of them were 100%) in several studies; additionally, our statistical analysis indicated a mean infection recurrence rate of up to 81.4% [9, 11, 19, 22, 23, 28, 29, 31, 37, 38]. This high failure rate could be attributed to the formation of biofilms by fungi, which diminished the efficacy of antimycotics when retaining the prosthesis. Del Pozo et al. [51] also listed FPJI as a contraindication for DAIR. Repeated DAIR not only put heavy psychological stress on patients, but also was more likely to cause damage and nonunion of soft tissues and skin around joints, affecting subsequent joint functions, such as knee extension. We suggested that DIAR should be used only in acute FPJI to alleviate severe clinical systoms, or as a preoperative preparation before staged exchange to improve the success rate of surgery, or as palliative treatment for patients who had serious basic diseases and were unable to endure complex surgery.

The treatment outcome of resection arthroplasty was not desirable, with an infection recurrence rate of 53.1%, which was a viable option for patients with low functional demands, limited mobility, insufficient bone structure, constraintof soft tissue quality, or a preference for no further treatment. In addition, salvage therapy should be considered only in cases of prolonged treatment failure or patient refusal of radical surgery. It is cruel to treat amputation or arthrodesis as the initial surgical interventions.

### Systemic and local antifungal therapy

Regardless of the surgical approach chosen, systemic antifungal treatment was the cornerstone of successful eradiction of FPJI. But the selection and duration of ideal antifungal drugs were more complicated and usually depended on the experience of surgeons and drug-sensitivity outcomes. No matter intravenous injection or oral administration, fluconazole was the most commonly used drug. Chronic suppression with fluconazole 400 mg daily was prescribed if prosthesis could not be removed [52]. The prevalence of azole-derived drugs resistance may limited its use, especially in Candida species. Other studies have also reported the utilization of echinocandins, such as caspofungin, and proved a feasible alternative for individuals who were unable to tolerate fluconazole or amphotericin B [19, 37, 38]. The debate about the ideal time of antimycotics administration was fierce. The duration of the report varied from one to 104 weeks. Infectious Diseases Society of America stongly advised that the antifungal drugs should last at least 12 weeks after resection arthroplasty and at least six weeks after reimplantation, whereas International Consensus Meeting recommended at least six weeks after resection arthroplasty. Kuo et al. [9] suggested that using antifungal drugs for six to 12 weeks after resection arthroplasty and six weeks to six months after reimplantation. A study identified improved infection eradication with prolonged systemic therapy from three to six months. However, Kuiper et al. [23] showed that patients who received the same antifungal drugs but different treatment time showed the same treatment results. Therefore, the successful eradiction of FPJI needed the cooperation of joint surgeons and infectious disease physicians, and personalized medicine according to microbiology and the status of patients. Additionally, it was imperative to assess the susceptibility of pathogens to antimycotics at different time intervals to prevent the potential alteration of drug sensitivity during treatment, which may resulted in treatment failure [27, 53].

The local drug concentration obtained from antifungal-loaded bone cement far exceeded that of systemic administration, while also demonstrating a systemical safe. The process of antibiotic elution can be characterized by two distinct phases: an initial rapid release within the first 7–10 days, followed by a gradual and continuous decline over a duration of four to six weeks [54]. Some clinical and experimental studies were conducted to explore the optimal characteristics of antimycotics incorporated into bone cement. Goss et al. [55] showed that amphotericin B had poor elution with less than 0.03% released after 1 week. This seemingly explained the finding of Ueng et al. that there was no statistical difference in treatment outcomes between patients who did and did not use amphotericin-loaded cement spacer [11]. Although the addition of high-dose poragen increased the elution of amphotericin B from bone cement, unforunately, compressive strength also decreased, which limited its clinincal use to fix implations [56]. It seemed that amphotericin B was not a good choice in the local application of FPJI. But liposomal amphotericin B exhibited a better performance in vivo and in vitro experiments, with a higher release from bone cement compared to amphotericin B [57, 58]. In addtiion, the utilization of voriconazole in cement spacers is experiencing a growing trend. Miller et al. [59] showed that the release rate of voriconazole-loaded bone cement was still more than 50% by Day 30. Denes et al. [60] also demonstrated that voriconazole exhibited stability and detectability in the presence of the exothermic reaction associated with cement polymerization. International Consensus Meeting highly recommened using liposomal amphotericin B or voriconazole impregnated cement spacers [61].

### Risk factors of recurrence 

We found that, in comparison to the hip joint, the knee joint was a protective factor against infection recurrence (OR = 0.424, *p* = 0.001). Brown et al. [28] reported that patients with fungal PJI of the hip have significantly lower infection-free survival rates. This scenario potentially arose from the more complex deridement of the hip joint, especially in acetabular side and the area near groin and abdomen, where fungal colonization can be found [37]. CA could generate a larger and more intricate biofilm than other species, thereby enhancing its resistance against antimycotics, [62], which explained why FPJI caused by CA increases the risk of infection recurrence in our systematic review (OR = 1.717, *p* = 0.041). Grzelecki et al. [35] and Karczewski et al. [34] concluded that success rate of the treatment was significantly lower in patients of PJI with CA than other fungi. Our analysis also showed that the use of antifungal agents after reimplantation and their application in spacer or cement were protective factors against infection recurrence (OR = 0.170, *p* = 0.002 and OR = 0.293, *p* = 0.020, respectively). In addition, previous studies demonstrated that several indicators, icluding the prosthesis-free interval, the mean length of antifungal treatment, patients with CCI ≥ 3, and CRP ≥ 6 mg/dL at the time of diagnosis, were potential risk factors for infection recurrence [38, 63]. Based on these available data, it was imperative to implement more proactive treatment measures and more closer postoperative re-examination where patients exhibited the aforesaid risk factors.

### Limitations

As a systematic review, there were some limitations that could not be avoided. First, inconsistency in information between manuscripts diminished the pool of events suitable for analysis and restrict the the value of statistical analysis. Not specific data in all patients was presented or provided different research indicators, necessitating the limitation of our regression analysis of risk factors for infection recurrence to univariate, which may wrongly caused certain variables to behave as risk or protective factors due to the presence of a confounder. Second, all articles included in this review were retrospective studies. It may cause bias in their data and further affect the results of our study, so articles with higher levels of evidence are essential. Third, we were unable to control variations in inclusion and exclusion criteria employed by authors of the included articles, despite our adherence to rigorous and objective criteria during the study selection process.

## Conclusions

Two-stage exchange remains the most commonly used surgical approach at present and combines with systemic antifungal treatment between stages and after reimplatation. Although the infection recurrence rate of one- and three-exchange is lower than that of two-exchange, its reliability needs further evaluation due to the small sample size. Antifungal-loaded cement spacers, and direct intra-articular injections of antimycotics after reimplatation should be strongly considered.Medication is not standardized but rather individualized according to microbiology and the status of patients, such as duration.

## Data Availability

The final dataset will be available from the corresponding author.

## Abbreviations

TJA: Total joint arthroplasty
FPJI: Fungal periprosthetic joint infection
CRP: C-reactive protein
ESR: Erythrocyte sedimentation rate
mNGS: Metagenomic next-generation sequencing
CA: Candida albicans
MSIS: Musculoskeletal Infection Society
BMI: Body mass index
ASA: American Society of Anaesthesiologists
CCI: Charlson Comorbidity Index
DAIR: Debridement, antibiotics, and retention of the implant
